# Black Raspberries Enhance Natural Killer Cell Infiltration into the Colon and Suppress the Progression of Colorectal Cancer

**DOI:** 10.3389/fimmu.2017.00997

**Published:** 2017-08-16

**Authors:** Pan Pan, Siwen Kang, Youwei Wang, Ka Liu, Kiyoko Oshima, Yi-Wen Huang, Jianying Zhang, Martha Yearsley, Jianhua Yu, Li-Shu Wang

**Affiliations:** ^1^Division of Hematology and Oncology, Department of Medicine, Medical College of Wisconsin, Milwaukee, WI, United States; ^2^Division of Hematology, Department of Internal Medicine, College of Medicine, The Ohio State University, Columbus, OH, United States; ^3^Department of Pathology, Medical College of Wisconsin, Milwaukee, WI, United States; ^4^Department of Obstetrics and Gynecology, Medical College of Wisconsin, Milwaukee, WI, United States; ^5^Center for Biostatistics, The Ohio State University, Columbus, OH, United States; ^6^Department of Pathology, The Ohio State University, Columbus, OH, United States; ^7^Comprehensive Cancer Center, The James Cancer Hospital, The Ohio State University, Columbus, OH, United States

**Keywords:** colorectal cancer, black raspberries, natural killer cells, immune surveillance, *Apc^Min/+^*, azoxymethane, dextran sulfate sodium

## Abstract

Natural killer (NK) cells are an essential component of innate immunity against cancer development. Many studies have been conducted to evaluate immune-modulating effects using dietary compounds. Our laboratory has been investigating the chemopreventive potential of black raspberries (BRBs) and previously demonstrated their beneficial modulation of genetic and epigenetic biomarkers in patients with colorectal cancer (CRC). The current study investigated their potential on modulating NK cells. To avoid the excessive inflammation caused by the dextran sulfate sodium (DSS) treatment that leads to colitis, we treated the mice with overnight DSS so that it would slightly irritate the colon but still promote colon carcinogenesis with 100% incidence in both the *Apc^Min/+^* mice and azoxymethane (AOM)-treated mice. A significant decrease of tissue-infiltrating NK cells along the progression of microadenoma-to-adenoma and adenoma-to-adenocarcinoma was observed in the *Apc^Min/+^*/DSS and AOM/DSS mice, respectively. Depletion of NK cells significantly promoted the development of CRC, suggesting a critical role of NK cells in combating CRC progression. BRBs significantly suppressed the CRC progression and increased the number of tissue-infiltrating NK cells in both mouse models. Moreover, we further determined BRBs’ effects on NK cells in the human biopsy specimens collected from our previously completed clinical trial, in which CRC patients consumed BRBs for an average of 4 weeks during a presurgical window. We observed an increased number and an enhanced cytotoxicity of NK cells by BRB intervention. The current study provides evidence that BRBs have the potential to enhance the tumor immunesurveillance of NK cells that can be beneficial in the setting of CRC prevention and treatment.

## Introduction

Colorectal cancer (CRC) is the third most common cancer in the world, with 54% of cases occurring in developed countries (1). A higher incidence of CRC is observed in Oceania and Europe whereas Africa and Asia have a lower incidence (1, 2). This higher incidence in developed countries can be, at least partially, attributable to the Western lifestyle of high intake of red meat and processed meat, which has been reported to be positively associated with a higher risk of CRC (3, 4). Therapeutic options depend on the stages when the patients are diagnosed. Patients at early stages with small tumors have a much better 5-year survival rate than the patients who are diagnosed at late stages (1). Intensive research has been conducted to search for biomarkers that could detect colonic polyps at early stages (5) that could, in turn, enhance the overall survival rate. Moreover, evidence has also emerged that CRC is largely preventable, if we eat and drink healthier, increase physical activities, and avoid obesity (6–9).

Dextran sulfate sodium (DSS) is an irritant that induces extensive colonic mucosal inflammation and it is frequently used to induce colitis. Cycles of DSS in drinking water promote colon tumors in *Apc^Min/+^* mice, where the incidence of adenoma is 100% and that of adenocarcinoma 22–100% by 10–12 weeks of age (10, 11). A single intraperitoneal (i.p.) injection of azoxymethane (AOM) followed by 4–7 days of DSS induces adenoma in 60–80% of C57/B6 mice and adenocarcinoma in 50% of them by 23 weeks of age, providing another colitis-associated CRC model (12). However, severe inflammation in these models is irrelevant to human sporadic CRC. Therefore, to avoid the excessive inflammation caused by cycles of DSS treatment that leads to colitis, we successfully manipulated the dose and duration of DSS so that it would only slightly irritate the colon but still promote colon carcinogenesis with 100% incidence in both the *Apc^Min/+^* mice and AOM-treated mice. These models pathologically and molecularly recapitulate human CRC that is promoted by mild inflammation, providing useful tools for studying this disease.

The host immune system plays a complex and multifaceted role in the development of CRC and patients’ responses to therapies (13). Natural killer (NK) cells are the innate arm of the immune system and are the first line of defense against cancer (14). NK cells express activating receptors, such as NK group 2 member D, DNAX accessory molecule-1, NKp46, NKp44, and NKp30, which recognize the ligands on the surface of tumor cells (15, 16). Decreased levels of the activating receptors have been observed in CRC patients (17–19). NK cells also express the activating receptor FcγRIIIa that binds to the immunoglobulin G1 to induce the antibody-dependent cellular cytotoxicity (16). NK cells also expression Fas ligand (FasL), which binds and activates death receptor Fas on tumor cells and induces apoptosis (20, 21). Furthermore, the cytokines produced by NK cells, including interferon-gamma (IFNγ) and chemokine (C–C motif) ligand 5, could have direct antitumoral effects (16, 22). Studies have reported that intra-tumoral NK cells are scarce in CRC tissues (23) and a greater infiltration of NK cells into the tumor tissues might be associated with a better prognosis (24, 25). Therefore, promoting the infiltration of NK cells and/or enhancing their cytotoxic function can be beneficial to CRC patients.

A strong link between diet and CRC has been elicited (6, 26) and many natural compounds have been identified by our laboratory and other groups as chemopreventive agents against CRC, such as black raspberries (BRBs) (27–30), resveratrol (31), and green tea extracts (32). A new field of nutritional immunology is emerging as more studies have been conducted to investigate the immune-modulating effects of natural compounds against CRC. For instance, one group treated *Apc^Min/+^* mice with 2% DSS in drinking water for 7 days, and the resulting colitis-associated CRC was associated with increased number of CD4^+^ T, CD8^+^ T cells, B cells, NK T cells, and myeloid-derived suppressing cells (33). Treatment with resveratrol protected against colon polyp development by reversing the DSS-induced inflammation and decreasing the inflammatory immune cells (33, 34). Another study showed that phyllanthusmin C, a plant-derived diphyllin lignan glycoside, enhanced IFNγ production by human NK cells (35). These findings suggest that natural compounds might combat CRC by regulating the immune system. The current study used tissues collected from our previously completed BRB intervention trial in CRC patients (27) as well as aforementioned *Apc^Min/+^*/overnight DSS and AOM/overnight DSS mouse models. This study showed that BRBs have strong potential to enhance the tumor immunesurveillance of NK cells.

## Materials and Methods

### Human Specimens

The present trial with CRC patients was approved by the Institutional Review Boards of the Ohio State University Comprehensive Cancer Center and the University of Texas, San Antonio. Inclusion criteria and exclusion criteria were described in our previous publication (27). All the patients who enrolled in this trial have provided written informed consent. Colon adenocarcinoma tissues of pre- and post-BRB intervention from nine patients who consumed BRBs for an average of 4 weeks were used in the current study.

### Animal, Diets, and Reagents

All animal experiments were approved by the Medical College of Wisconsin Animal Care and Use Committee. Eight-week-old breeding pairs of wild-type (WT) and *Apc^Min/+^* mice were purchased from the Jackson Laboratory (Bar Harbor, ME, USA).

The control diet, the American Institute of Nutrition (AIN)-76A, was purchased from Dyets Inc. (Bethlehem, PA, USA). The BRB powder was purchased from Berri Products (Corvallis, OR, USA) and stored at 4°C in vacuum-sealed plastic bags at the Medical College of Wisconsin. AOM was obtained from Sigma (St. Louis, MO, USA) and dextran sulfate sodium (DSS, 36,000–50,000 M.W.) was obtained from MP Biochemicals (Santa Ana, CA, USA).

### Cell Lines

CT26 (CRL-2638), GS-109-V-63 cells (CRL-1614), and K-562 cells (CCL-243) were purchased from the American Type Culture Collection (ATCC, Manassas, VA, USA). These cells were cultured as recommended and cryopreserved in liquid nitrogen. The cells were not re-authenticated as they were passaged for fewer than 6 months after resuscitation. VACO-235 and VACO-330 cells were kind gifts from Dr. Sanford Markowitz at the Case Western Reserve University. These cells were cultured as previously described (36, 37).

### Animal Experiments

Four-to-five-week-old *Apc^Min/+^* mice were given the AIN-76A control diet. These mice were fed with 5% DSS in their drinking water overnight (at week 1). Two weeks after the DSS treatment (at week 3), subgroups of the *Apc^Min/+^*/DSS mice were changed to the diet supplemented with 5% BRBs for 4 weeks. Six weeks after the DSS treatment (at week 7), all the *Apc^Min/+^*/DSS mice were euthanized by CO_2_ asphyxiation. The colonic polyp number and size were determined.

Five- to six-week-old WT mice were given the AIN-76A control diet and injected intraperitoneally with AOM (15 mg/kg body weight) (at week 1). One week after the injection, the mice were given 5% DSS in their drinking water overnight (at week 2). Two weeks after the DSS treatment (at week 4), subgroups of the AOM/DSS mice were changed to the diet supplemented with 5% BRBs for 4 weeks. Six weeks after the DSS treatment (at week 8), all the AOM/DSS mice were euthanized by CO_2_ asphyxiation. The colonic polyp number and size were determined.

Colons from all mice were collected, fixed in formalin, and embedded in paraffin (FFPE). Hematoxylin and eosin (H&E) staining was performed. Stained slides were photographed at 20× magnification using an Olympus microscope. The tissue sections were examined by certified pathologists. The entire colon was viewed under high power magnification (20×). The percentage of high-grade dysplasia, adenoma, or adenocarcinoma was calculated as lesion areas over total areas of the entire length of the colon.

### Depletion of NK Cells

Two weeks after the DSS treatment, subgroups of the *Apc^Min/+^*/DSS mice and AOM/DSS mice were injected intraperitoneally with anti-NK1.1 antibodies (0.2 mg, Bio X Cell, West Lebanon, NH, USA) once per week for 4 weeks.

### Immunohistochemistry

FFPE colon tissue blocks were cut into 4 µm sections and immunohistochemistry was conducted as previously described (30). A Dako Link 48 Autostainer Immunostaining System (Agilent Technologies, Santa Clara, CA, USA) was used to stain the slides with primary antibodies to CD56 (NCL-L-CD56-1B6) from Leica Biosystems (Buffalo Grove, IL, USA), and primary antibodies to NCR1 (NKp46, bs-10027R), and LAMP1 (CD107a, bs-1970R) from Bioss (Woburn, MA, USA). Human colon tissues were stained with the EnVision G2 Doublestain System (K536111-2, Agilent Technologies) using anti-CD56 antibodies linked to Vina Green Chromogen (BRR807AH, Biocare Medical, Concord, CA, USA) and anti-LAMP1 antibodies linked to Permanent Red Chromogen. The staining pattern of LAMP1 was similar to the representative images shown on the antibody company website using human small intestine (38). Stained slides were photographed at 20× magnification and only the staining in the adenoma and adenocarcinoma area was quantified as previously described (30). Specificity of each antibody had been confirmed and no positive staining was observed when using control isotype.

### Colonic Lamina Propria (LP) Preparation

Colonic LP samples were prepared by a Dissociation Kit (130-097-410) from Miltenyi Biotec (San Diego, CA, USA) as previously described (39). Briefly, an independent cohort of the *Apc^Min/+^* mice treated with overnight DSS and WT mice treated with an AOM injection as well as overnight DSS were fed either the AIN-76A control diet or the diet supplemented with the 5% BRBs 2 weeks after the DSS treatment for 4 weeks. All the mice were euthanized by CO_2_. Colonic specimens were collected, washed, enzymatically digested using Digestion Solution and Enzyme Mix, and further mechanically broken by gentleMACS™ Dissociator (Miltenyi Biotec). Digested tissue samples in the gentleMACS™ C tubes were centrifuged to form a cell pellet, followed by re-suspending in DMEM for flow cytometry.

### Flow Cytometry

Mouse colonic LP samples were prepared for staining with anti-mouse CD45, CD3, NKp46, CD107a, IFNγ, and CD178 (FasL) antibodies (BD Biosciences, Franklin Lakes, NJ, USA). Human cell lines K-562, VACO-235, and GS-109-V-63 were prepared for staining with antihuman major histocompatibility complex (MHC) class I (HLA-A, B, C) antibodies (Biolegend, San Diego, CA, USA). The samples were analyzed by a LSRII flow cytometer (BD Biosciences), and FlowJo software (Tree Star, Ashland, OR, USA) was used to analyze the data.

### Standard ^51^Cr Release Cytotoxicity Assay

Primary human NK cells were isolated from fresh peripheral blood leukopaks (American Red Cross, Columbus, OH, USA) as described previously (40). Murine splenic NK cells were isolated from fresh mouse spleen. A standard 4-h ^51^Cr release assay was performed as described previously (41). Briefly, target cells were labeled with ^51^Cr and co-cultured with NK cells at various effector/target ratios (E/T) in the wells of 96-well V-bottom plates at 37°C for 4 h. Supernatants were harvested and transferred into scintillation vials containing a liquid scintillation cocktail (Fisher Scientific, Waltham, MA, USA), and the release of ^51^Cr was measured on Beckman Liquid Scintillation Counter LS-6500. Target cells incubated in complete medium or 1% SDS were used to determine spontaneous or maximal ^51^Cr release, respectively. Percentage of specific cell lysis was calculated using the standard formula: 100 × (cpm experimental release − cpm spontaneous release)/(cpm maximal release − cpm spontaneous release).

### Statistical Analysis

Using SigmaPlot (Systat Software, San Jose, CA, USA), we performed unpaired two-tailed Student’s *t*-tests to determine the changes at different stages (the early stage versus the late stage) and the dietary effects (the control diet versus the 5% BRB diet). A *p-*value less than 0.05 was considered statistically significant.

## Results

### Infiltration of NK Cells Decreases from Microadenoma to Adenoma in *Apc^Min/+^*/DSS Mice, and BRBs Suppress the Progression of Colon Tumorigenesis and Enhance Tumor-Infiltrating NK Cells

**Figure 1:**
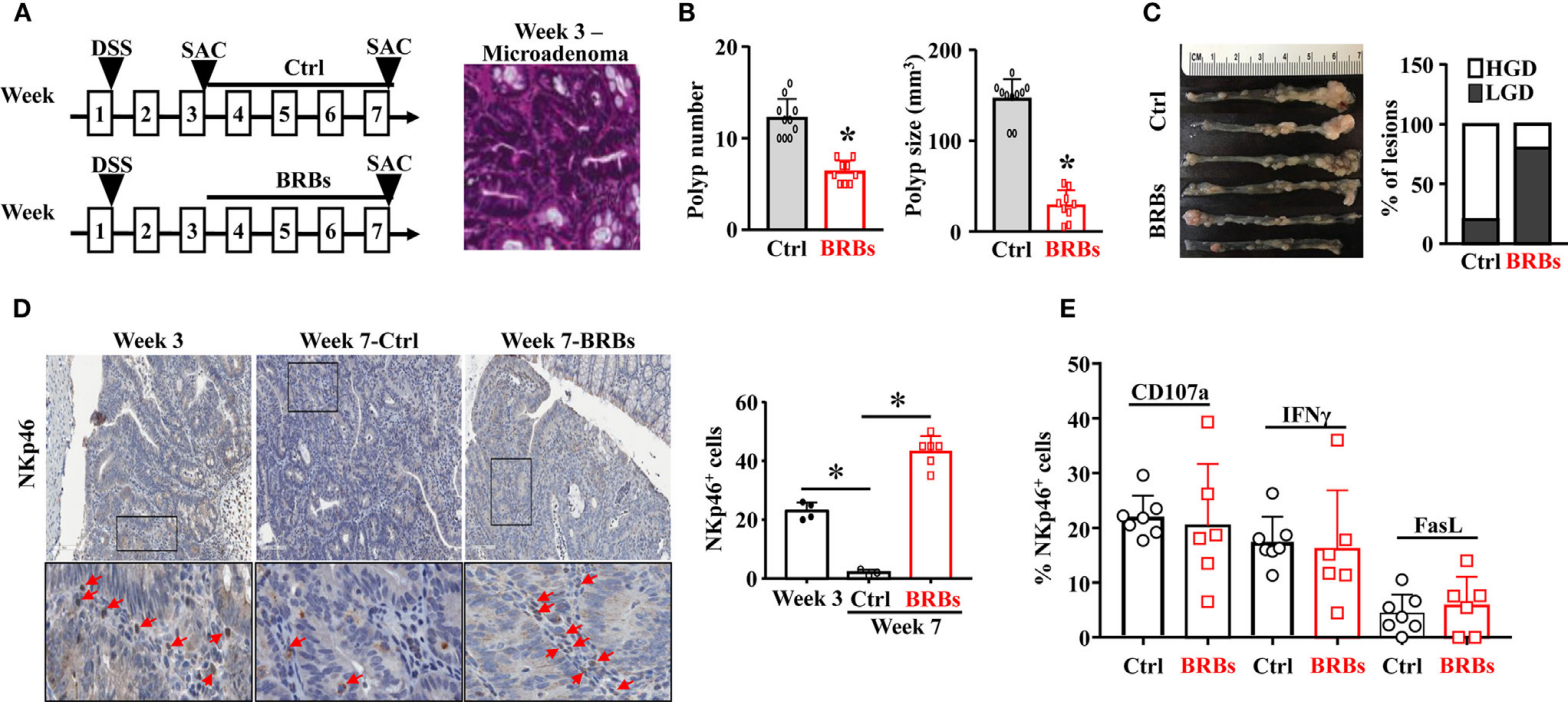
Black raspberries (BRBs) enhance tumor-infiltrating natural killer cells and suppress the progression of microadenoma to adenoma in *Apc^Min/+^*/DSS mice. **(A)** *Apc^Min/+^* mice were treated with 5% DSS in the drinking water overnight, fed the control diet, and euthanized at week 3 and week 7. Subgroups of the *Apc^Min/+^*/DSS mice were fed the 5% BRBs from week 3 and euthanized at week 7. The colon tissues were collected and H&E staining were performed. **(B)** The colonic polyp number and size of the *Apc^Min/+^*/DSS mice were examined. **(C)** Histopathologic evaluations of colon lesions. **(D)** The colon tissues were stained with anti-NKp46 antibodies; the staining in the adenoma areas was quantified. **(E)** Colonic LP samples were used to measure the percentage of CD107a^+^, IFNγ^+^, or Fas Ligand^+^ (FasL)^+^ cells by flow cytometry. HGD: high-grade dysplasia; LGD: low-grade dysplasia. *p < 0.05.

To recapitulate human familial adenomatous polyposis, we treated the *Apc^Min/+^* mice with 5% DSS in the drinking water overnight to slightly irritate the colon. The *Apc^Min/+^*/DSS mice were given the control diet and were euthanized at week 3 and week 7. Subgroups of the *Apc^Min/+^*/DSS mice were given the BRB diet from week 3 and were euthanized at week 7 (Figure 1A). The colon tissues were collected to examine the polyp number and size, as well as the histopathological diagnosis of polyp lesions. Microadenoma occurred in the colon at week 3 (Figure 1A). The incidence of colonic polyp in the control diet-treated *Apc^Min/+^*/DSS mice was 100% by week 7. The BRB-treated *Apc^Min/+^*/DSS mice developed significantly fewer and smaller colonic polyps compared to mice that were fed the control diet (Figure 1B). Also, the polyps of BRB-treated *Apc^Min/+^*/DSS mice presented a smaller proportion of high-grade dysplasia whereas those of control diet-fed *Apc^Min/+^*/DSS mice presented a higher proportion of high-grade dysplasia (Figure 1C).

In order to determine the infiltration of NK cells into the colonic polyps, we stained the colon tissues with anti-NKp46 antibodies. We found that significantly fewer NK cells infiltrating into colon polyps at week 7 compared to week 3, and BRBs significantly increased the tissue-infiltrating NK cells at week 7 (Figure 1D). To characterize the functions of NK cells, flow cytometric analysis was taken to measure the levels of CD107a, a functional marker for NK cells’ cytotoxic activities (42), IFNγ production (16, 22), and FasL expression in NK cells of colonic LP samples. The levels of CD107a^+^, IFNγ^+^, and FasL^+^ cells in colonic LP were comparable between the control and BRB diet group (Figure 1E), suggesting that a higher number of the infiltrating NK cells into colon tissues contributes to the BRBs’ protective effects in *Apc^Min/+^*/DSS mice.

### Infiltration of NK Cells Decreases from Adenoma to Adenocarcinoma in AOM/DSS Mice, and BRBs Suppress the Progression of Colon Tumorigenesis and Enhance Tumor-Infiltrating NK Cells

**Figure 2:**
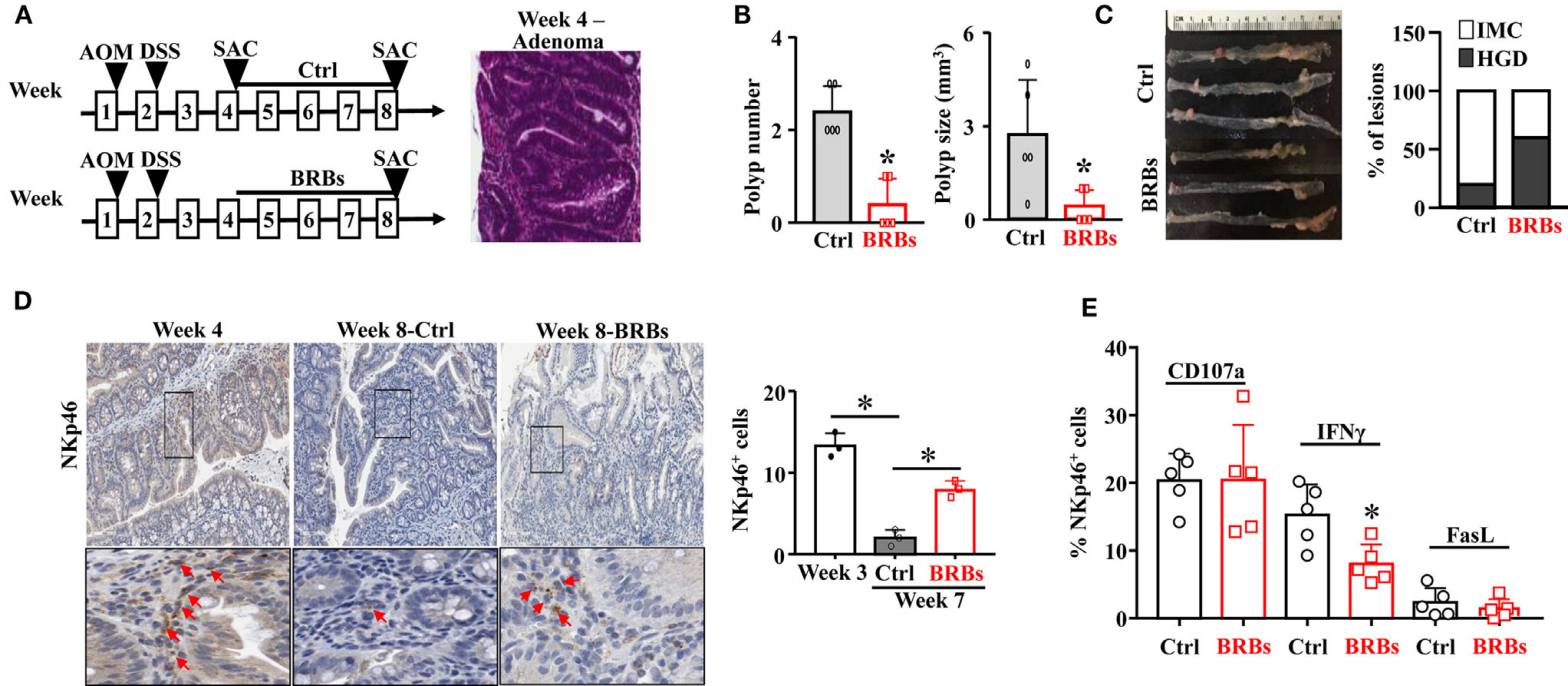
Black raspberries (BRBs) enhance tumor-infiltrating natural killer cells and suppress the progression of adenoma to adenocarcinoma in azoxymethane (AOM)/DSS mice. **(A)** WT mice were treated with one dose of AOM (15 mg/kg body weight, i.p.) followed by 5% DSS in the drinking water overnight, fed the control diet, and euthanized at week 4 and week 8. Subgroups of the AOM/DSS mice were fed the 5% BRBs from week 4 and euthanized at week 8. **(B)** The colonic polyp number and size of the AOM/DSS mice were examined. **(C)** Histopathologic evaluations of colon lesions. **(D)** The colon tissues were stained with anti-NKp46 antibodies; the staining in the adenoma and adenocarcinoma areas was quantified. **(E)** Colonic LP samples were used to measure the percentage of CD107a^+^, IFNγ^+^, or Fas Ligand^+^ (FasL^+^) cells by flow cytometry. IMC: intramucosal carcinoma; HGD: high-grade dysplasia. *p < 0.05.

In another mouse model to recapitulate human sporadic CRC, WT mice were treated with a single dose of AOM (15 mg/kg body weight) followed by 5% DSS in the drinking water overnight. The AOM/DSS mice were given the control diet and were euthanized at weeks 4 and 8. Subgroups of the AOM/DSS mice were given the BRB diet from week 4 and were euthanized at week 8 (Figure 2A). The colon tissues were collected to examine the polyp number and size, as well as the histopathological diagnosis of polyp lesions. The AOM/DSS mice developed adenoma at week 4 (Figure 2A) and 100% of these mice developed adenocarcinoma at week 8. Consistent with the *Apc^Min/+^*/DSS mouse model, 5% BRBs significantly suppressed the colonic polyp development (Figure 2B). The polyps from the BRB-treated AOM/DSS mice showed a lower percentage of intramucosal carcinoma in comparison to the control diet-fed AOM/DSS mice (Figure 2C).

Importantly, we observed a significantly decreased NK cell infiltration in the control diet-treated AOM/DSS mice at week 8 compared to those at week 4. BRBs significantly increased infiltration of NK cells into the polyps (Figure 2D). Neither CD107a nor FasL was significantly changed in colonic LP (Figure 2E). Interestingly, we observed a significantly decreased level of IFNγ after BRB treatment (Figure 2E). Collectively, our results suggest that infiltration of NK cells decreases along the progression from microadenoma to adenoma and further to adenocarcinoma, and BRBs enhance the tumor-infiltration NK cells at different stages of CRC progression.

### Depletion of NK Cells Exacerbates the Progression of CRC

**Figure 3:**
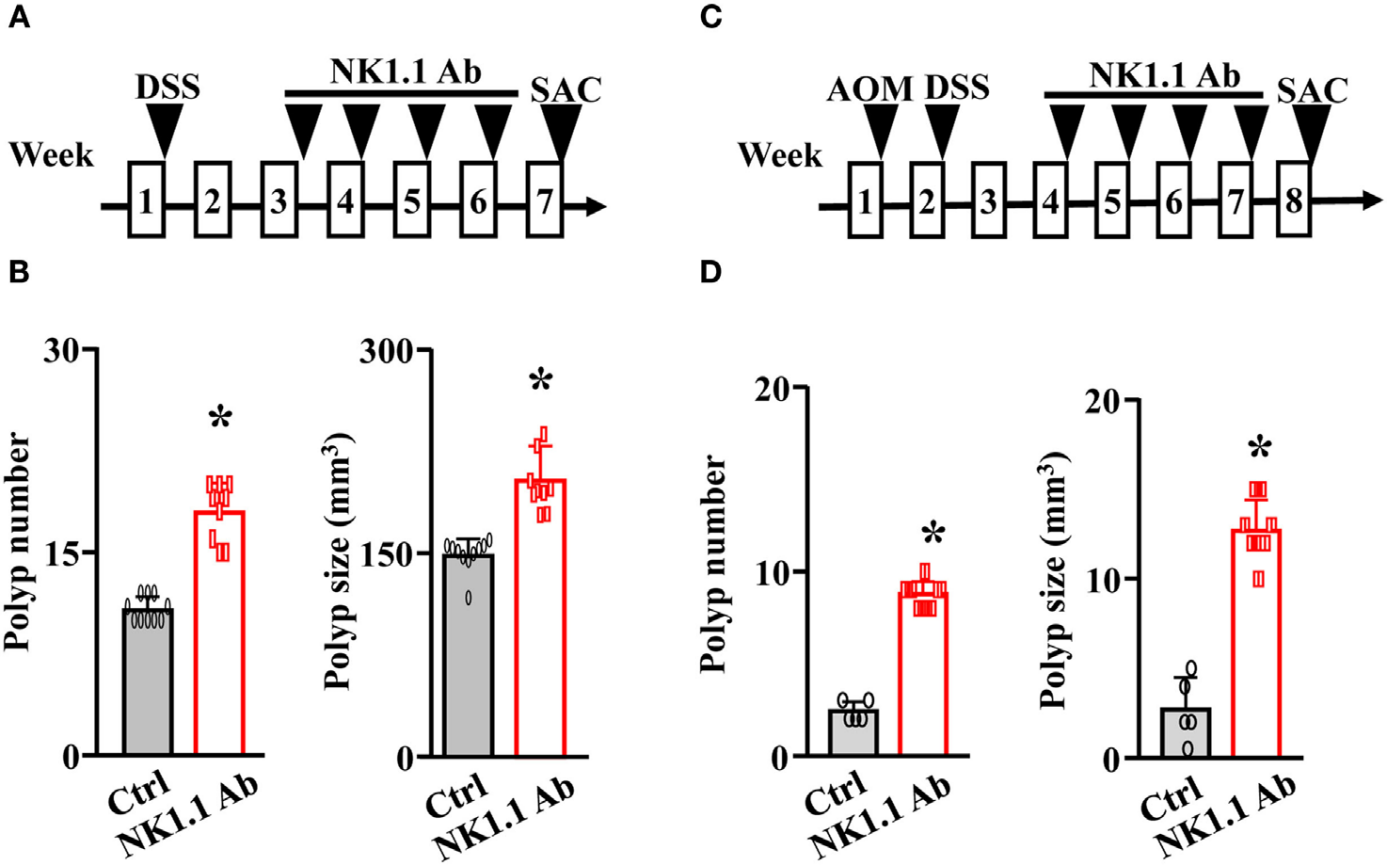
Depletion of natural killer cells exacerbates the progression of colorectal cancer. **(A)** The *Apc^Min/+^* mice were treated with 5% DSS overnight. Two weeks later, these mice were given either control IgG or anti-NK1.1 antibodies for 4 weeks. These mice were euthanized at week 7. **(B)** The colonic polyp number and size of the *Apc^Min/+^*/DSS mice were examined. **(C)** WT mice were treated with a single dose of azoxymethane (AOM) followed by 5% DSS overnight. Two weeks later, these mice were given either control IgG or anti-NK1.1 antibodies for 4 weeks. These mice were euthanized at week 8. **(D)** The colonic polyp number and size of the AOM/DSS mice were examined. *p < 0.05.

To further investigate the antitumorigenic role of NK cells in CRC, we depleted NK cells in both the *Apc^Min/+^*/DSS mouse model and the AOM/DSS mouse model using anti-NK1.1 antibodies (Figures 3A,C). Without the immune-surveillance of NK cells, these mice developed significantly more and larger colonic polyps (Figures 3B,D).

**Figure 4:**
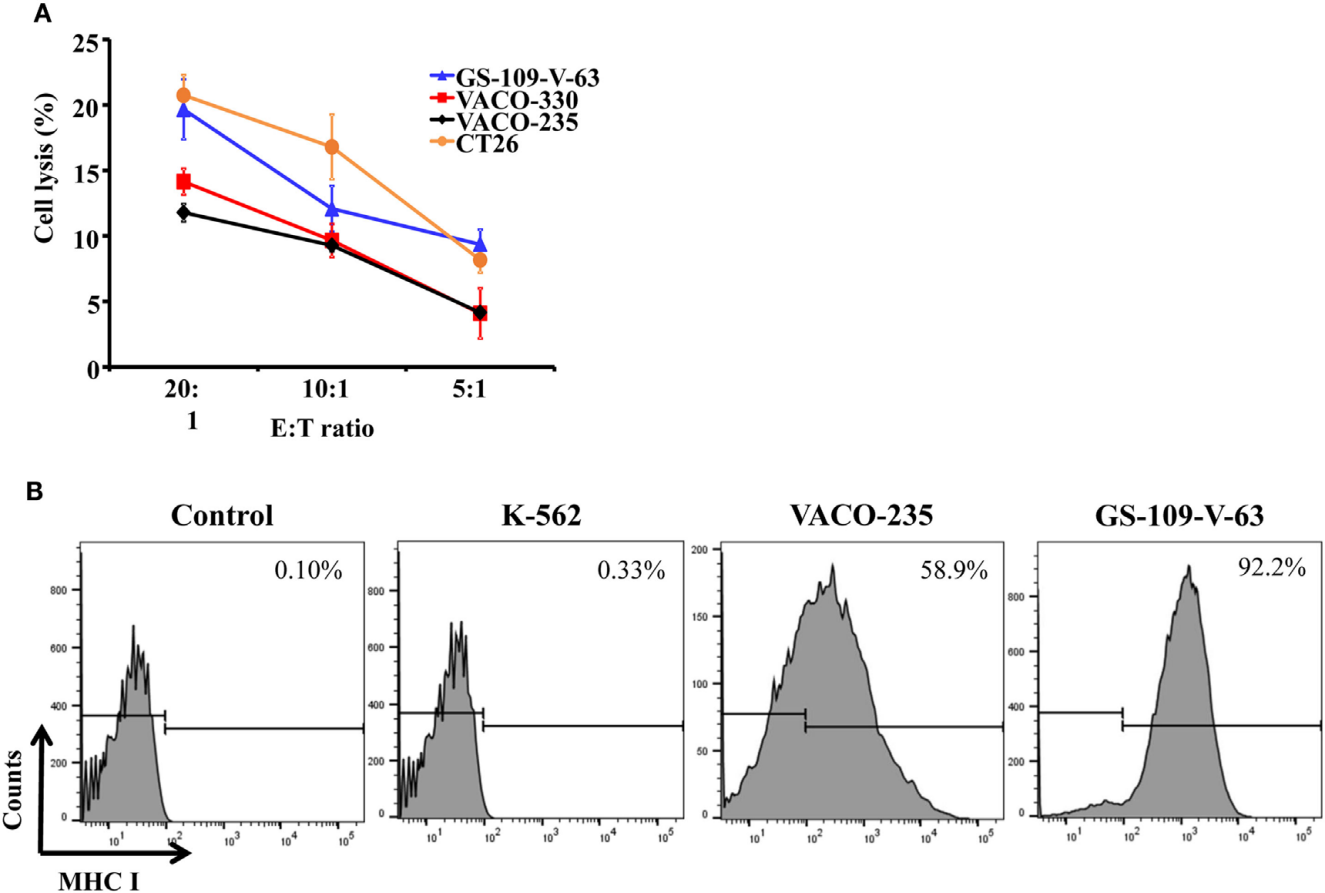
Primary natural killer (NK) cells have the capacity of lysing human precancerous cells and mouse carcinoma cells. **(A)** Human colon adenoma cells from familial adenomatous polyposis patients (GS-109-V-63), human advanced adenoma cells (VACO-235 and VACO-330), and mouse colon carcinoma cells (CT26) were used to determine the cytotoxicity of primary NK cells. **(B)** Human chronic myelogenous leukemia cell line (K-562), VACO-235, and GS-109-V-63 cells were used to determine the expression of major histocompatibility complex (MHC) class I molecules by flow cytometry.

In addition, we cocultured the primary mouse NK cells with mouse colon carcinoma cells, CT26, and showed that NK cells could lyse CT26 cells (Figure 4A). Also, we showed that the primary human NK cells could kill precancerous colon adenoma cells by using GS-109-V-63 cells, which were derived from patients with the Gardner’s syndrome, a form of familial adenomatous polyposis, and using VACO-235 and VACO-330 cells that were derived from patients diagnosed with villous and tubular adenoma in colon (36, 37) (Figure 4A). Using flow cytometry, we further determined the expression of MHC class I molecules, which serve as inhibitory ligands for NK cells, on the surface of human precancerous colon adenoma cells. We observed a substantially higher expression of MHC class I on the surface of VACO-235 (178-fold higher) and GS-109-V-63 (279-fold higher) cells compared with K-562 cells, which are sensitive to NK cells’ killing (Figure 4B). Therefore, these results suggest that NK cells are able to kill both the precancerous and cancerous cells, although the level of effector cell cytotoxicity is moderate due to high levels of the expression of MHC class I molecules in target cells.

### BRBs Increase Tumor-Infiltrating NK Cells in Human CRC Patients

**Figure 5:**
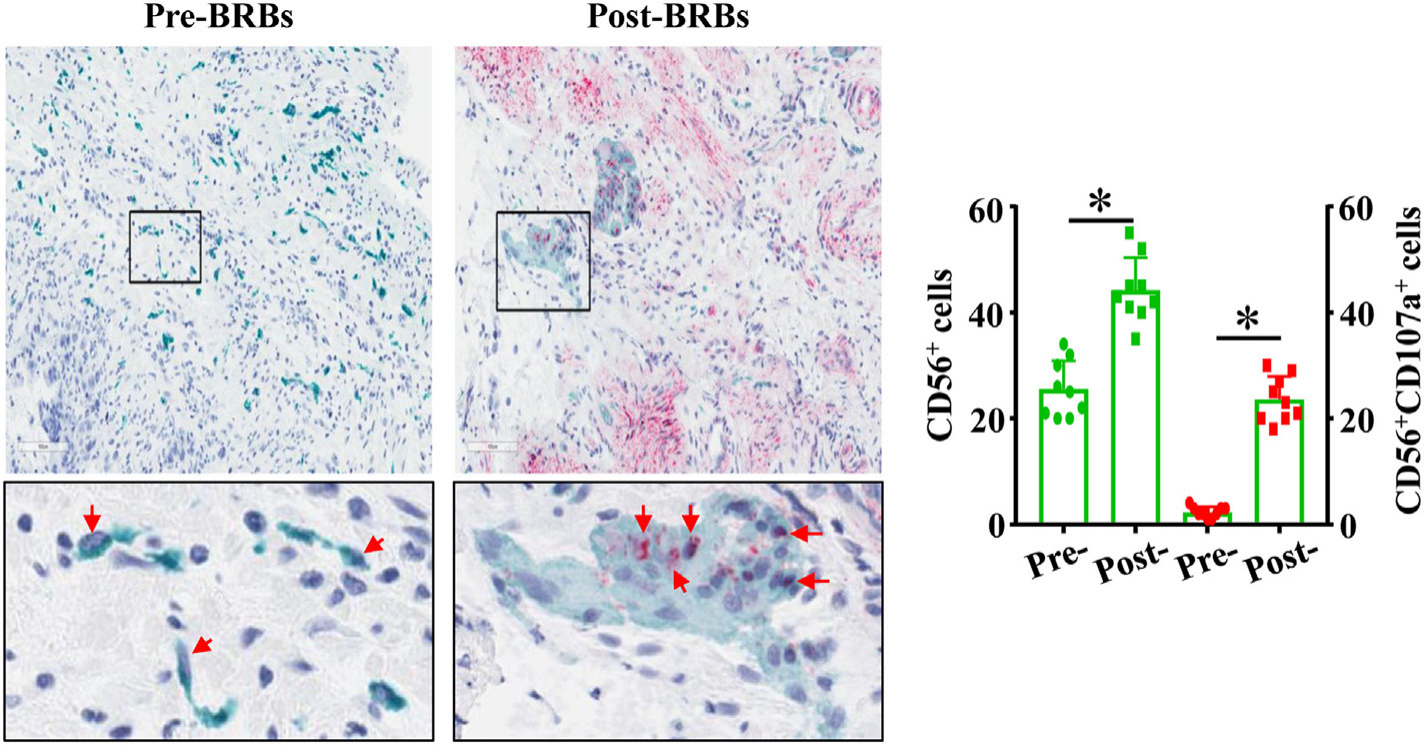
Black raspberries (BRB) intervention enhances the number and function of natural killer (NK) cells in colorectal cancer (CRC) patients. The pre-BRB and post-BRB colon biopsy specimens from nine CRC patients were double-stained with anti-CD56 (green staining) and anti-CD107a (red staining) antibodies. BRBs significantly increased the tumor-infiltrating NK cells in CRC patients. *p < 0.05.

Our previously completed human clinic trial demonstrated the beneficial effects of BRBs for CRC patients (27). Double-staining was performed on the colon biopsy tissues from this trial using anti-CD56 antibodies and anti-CD107a antibodies. We observed significantly increased levels of tumor-infiltrating NK cells (CD56^+^) and enhanced cytotoxic activities of these NK cells (CD56^+^CD107a^+^) in the post-BRB specimens compared to the pre-BRB specimens from the CRC patients (Figure 5). These results suggest a role of tumor-infiltrating NK cells in BRB-mediated chemoprevention in human CRC.

## Discussion

First proposed by Sporn and Newton in 1979 (43), chemoprevention is to utilize the natural, synthetic, or biologic agents that are able to delay, reverse, or inhibit tumorigenesis. Along with the advancing therapeutic strategies, cancer prevention and cancer interception can be a new promising era for cancer patients and more importantly for the patients with precancerous lesions and the population at high risk of cancer (44–47). CRC progresses in a well-defined adenoma–carcinoma sequence, which provides a good model to study chemoprevention at early stages of the disease by natural components. Our laboratories focused on CRC chemoprevention using dietary BRBs. Previously, we demonstrated that BRBs epigenetically modulated the expression of genes associated with the Wnt pathway, proliferation, and apoptosis in the human CRC patients (27) and familial adenomatous polyposis patients (28). BRBs could also modulate the biochemical metabolites that were associated with tumor proliferation and apoptosis in humans (29) and mice (30, 39, 48). Furthermore, the current study investigated their immune-modulating effects in the CRC mouse models, especially the effects on NK cells.

DSS is an irritant that induces extensive colonic mucosal inflammation. Altamemi et al. treated *Apc^Min/+^* mice with 2% DSS for 1 week to induce colitis and enhance polyp development. They observed significantly increased levels of inflammatory CD4^+^ T, CD8^+^ T cells, B cells, NK T cells, and myeloid-derived suppressing cells in mesenteric lymph nodes of these polyp-bearing mice compared to control mice. In addition, they observed significantly increased levels of pro-inflammatory cytokines, such as interleukin-6 and tumor necrosis factor-alpha, suggesting a strong inflammatory response occurred in these *Apc^Min/+^* mice treated with 1-week DSS. These changes were corrected by resveratrol-mediated anti-inflammatory effects (33). In addition, one group demonstrated that a population of regulatory NK T cells locally promoted intestinal polyp development by increasing regulatory T cells and suppressing the antitumor T-helper immunity in the intestinal polyps of *Apc^Min/+^* mice (49). Another study injected C57/B6 mice with a single dose of AOM and treated the mice with three cycles of DSS. They observed significantly increased levels of granulocyte colony-stimulating factor, a pro-inflammatory cytokine, in colon organ culture supernatant. Administration of anti-granulocyte colony-stimulating factor antibodies suppressed the colon carcinogenesis and significantly increased the levels of NK cells and IFNγ-producing CD4^+^ and CD8^+^ T cells (50). Compared with other studies, our study is unique that we treated *Apc^Min/+^* mice with 5% DSS only overnight that only caused mild inflammation in the colon. Colon tumors were presented 6 weeks later, and we observed significantly decreased NK cells in colon tumors, which were reversed by BRBs. These results suggest that colonic inflammation and altered immune cells’ tumor surveillance, as well as the dynamic interaction between these two factors, contribute to the development of CRC. The degrees of colonic inflammation and whether the studies were conducted in tumor-bearing animals lead to different immune cell profiling in different studies.

The concept of “cancer immune-editing” has been proposed to constitute in both cancer initiation and progression through immune suppression or escape (51). Tumor cells develop various mechanisms in the process of immune escape, such as downregulating the presentation of ligands recognized by NK cells or cytotoxic T cells, and/or promoting tumor-enhancing cytokines. NK cells are important components of innate defense against neoplastic cells and viruses. Altered MHC class I expression, NK activating receptors, or Fas-FasL activation could all compromise NK cells’ tumor surveillance (51, 52). Studies have investigated the clinical impact of NK cell infiltration on the prognosis of CRC (53). However, the findings were inconsistent between studies: several groups reported a favorable prognostic impact with higher NK infiltration (24, 54); while others found no effect on the clinical development of CRC (55). Nonetheless, one group suggested that the presence of both NK cells and CD8^+^ T cells in the tumor microenvironment of CRC may result in a favorable prognosis (25). In addition, cancer stem cells have been suggested to be the mechanism of metastasis of solid tumors (56). Although the low level of MHC class I expression on the cancer stem cells indicates a low efficiency of being targeted by CD8^+^ T cells, studies have demonstrated that NK cells are capable of killing cancer stem cells. For instance, one group observed a robust cytotoxic effect of NK cells against CRC-derived stem cells, compared to CRC cells (57, 58). This was associated with a higher expression of NCR ligands (NKp30 and NKp44) and a lower level of MHC class I on the surface of cancer stem cells (57). In the current study, we found a moderate cytotoxicity of NK cells against the human precancerous adenoma cells (VACO-235 and GS-109-V-63), which was likely due to the extremely high expression of MHC class I molecules on the surface of these cells.

In the current study, we showed a decrease of NK cell infiltration in the colonic polyps along the progression from microadenoma to adenoma and further to adenocarcinoma in mouse models. In addition, we depleted NK cells using anti-NK1.1 antibodies at the early stage in both mouse models and observed significantly promoted CRC progression. These results suggest that reduced NK cells may already be present at early stage of CRC, and this can be a critical window for chemopreventive agents to sustain or boost up NK cell population/function in cancer stages, as NK cells may have been altered in precancerous stages. Therefore, NK cells could play a role in the colon cancer progression.

We fed the mice with either the AIN-76A control diet or the 5% BRB diet when precancerous lesions occurred. We observed that BRB-fed mice developed smaller and fewer colon lesions with lower histopathological grades. Importantly, BRBs sustained/enhanced the number of tissue-infiltrating NK cells into the colonic polyps in both mouse models. We observed comparable expression of CD107a and FasL in NK cells of colonic LP, suggesting BRBs may not enhance the activities of NK cells in these two mouse models. Interestingly, we observed a decreased production of IFNγ using flow cytometry after BRB treatment in AOM/DSS model. Future studies would be needed to investigate if BRB-enhanced NK infiltration was a compensatory mechanism to rescue the decreased IFNγ production, in order to maintain the tumor surveillance of NK cells. Alternatively, the decreased IFNγ may lead to a low level of programmed death-ligand 1 expression on tumor cells, contributing to the reverse of the exhaustion of CD8^+^ T cells in the tumor microenvironment because IFNγ is a key factor that stimulates programmed death-ligand 1 expression (59).

Furthermore, in order to evaluate BRBs’ ability on modulating human NK cells, we stained the human biopsy specimens from our previously finished human clinic trial with anti-CD56 antibodies and anti-CD107a antibodies. This is the first study to measure the presence of infiltrated NK cells and their degranulation marker, CD107a, *in situ* in the tumor microenvironment with intervention by natural products. We observed a significant increase in the number and the CD107a expression of NK cells in CRC patients who consumed BRB diets. These results strongly suggest that BRB-mediated promotion in NK cell tumor infiltration and function can enhance tumor immune surveillance, thereby contributes to the chemopreventive effects of BRBs against CRC in humans. Future studies would be needed to investigate how BRBs promote both NK cell infiltration and function in CRC patients, while we only observed a higher NK cell infiltration, but not the function, in mice.

With the recognition of chemopreventive effects of berries, several studies have been conducted to investigate the effects of berries on NK cells. Our previous studies showed that BRBs increased the expression of interleukin-12, a cytokine that activates both cytolytic NK cells and CD8^+^ T cells in rodent esophageal cancer (60). In addition, one group reported that 6-week administration of blueberry power (equivalent to 250 g fresh berries) daily increased NK cell counts in the circulating blood compared to their pre-berry NK cell counts in the blood, while placebo power had no effect on the circulating NK cells (61, 62). Another group conducted a randomized, double-blind, placebo-controlled clinical trial with 60 older healthy Chinese adults and gave them 150 g Goji berries daily for 30 days. However, the number of NK cells was not significantly altered compared to placebo group in this trial (63). These results suggest that different berries may have different effects. However, these studies only investigated the effects of berries on circulating NK cell count in healthy populations. Our current study is novel, and it is the first study to indicate that BRBs have a potential to modulate NK cell infiltration and function against CRC in mice and humans.

In summary, our findings demonstrate the potential role of NK cells in the development of CRC in both mouse models and human CRC patients. BRBs have the potential to enhance tumor immune surveillance by promoting NK cell tumor infiltration and functions that can be beneficial in the setting of CRC prevention and treatment.

## Ethics Statement

All protocols followed with institutional guidelines for animal care dictated by the Medical College of Wisconsin Animal Care and Use Committee.

## Author Contributions

Study design, acquisition of data, analysis, and interpretation of data: PP, SK, YW, KL, KO, Y-WH, JZ, MY, JY, and L-SW. Manuscript writing and revising: PP, L-SW, and JY. Final approval: JY and L-SW.

## Conflict of Interest Statement

The authors declare that the research was conducted in the absence of any commercial or financial relationships that could be construed as a potential conflict of interest.

## Abbreviations

AOM, azoxymethane; AIN, American Institute of Nutrition; BRBs, black raspberries; CRC, colorectal cancer; DSS, dextran sulfate sodium; IFNγ, interferon-gamma; LP, lamina propria; NK, natural killer; WT, wild type.
